# The Duration of Motor Responses Evoked with Intracortical Microstimulation in Rats Is Primarily Modulated by Stimulus Amplitude and Train Duration

**DOI:** 10.1371/journal.pone.0159441

**Published:** 2016-07-21

**Authors:** Meghan Watson, Mohamad Sawan, Numa Dancause

**Affiliations:** 1 Polystim Neurotechnologies, Institute of Biomedical Engineering, Polytechnique, Montreal, Quebec, Canada; 2 Département de Neurosciences, Faculté de Médecine, Université de Montréal, Montreal, Quebec, Canada; 3 Groupe de Recherche sur le Système Nerveux Central (GRSNC), Université de Montréal, Montreal, Quebec, Canada; Duke University, UNITED STATES

## Abstract

Microstimulation of brain tissue plays a key role in a variety of sensory prosthetics, clinical therapies and research applications, however the effects of stimulation parameters on the responses they evoke remain widely unknown. In particular, the effects of parameters when delivered in the form of a stimulus train as opposed to a single pulse are not well understood despite the prevalence of stimulus train use. We aimed to investigate the contribution of each parameter of a stimulus train to the duration of the motor responses they evoke in forelimb muscles. We used constant-current, biphasic, square wave pulse trains in acute terminal experiments under ketamine anaesthesia. Stimulation parameters were systematically tested in a pair-wise fashion in the caudal forelimb region of the motor cortex in 7 Sprague-Dawley rats while motor evoked potential (MEP) recordings from the forelimb were used to quantify the influence of each parameter in the train. Stimulus amplitude and train duration were shown to be the dominant parameters responsible for increasing the total duration of the MEP, while interphase interval had no effect. Increasing stimulus frequency from 100–200 Hz or pulse duration from 0.18–0.34 ms were also effective methods of extending response durations. Response duration was strongly correlated with peak time and amplitude. Our findings suggest that motor cortex intracortical microstimulations are often conducted at a higher frequency rate and longer train duration than necessary to evoke maximal response duration. We demonstrated that the temporal properties of the evoked response can be both predicted by certain response metrics and modulated via alterations to the stimulation signal parameters.

## Introduction

Since its advent in the early 19th century, stimulation of the brain has been used in a wide variety of clinical and therapeutic applications, many of which involve novel treatments for diseases and disorders such as visual [1–4] and somatosensory [5–7] prosthetic devices, and deep brain stimulation therapies for Parkinson’s disease [8–11] and epilepsy [12–14]. These applications inject an electrical stimulus into neural circuitry in order to modify activity or produce sensations or behaviors. While brain stimulation plays a crucial role in countless therapies and research areas, little is known about how the parameters of the stimulation signal influence neural activity or how they shape the outputs produced.

Many types of stimulation signal have been explored, but the most prevalent is the constant-current, cathode leading, biphasic square waveform [15]. The parameters of this signal include the current amplitude, pulse frequency, pulse duration, interphase interval and pulse train duration. Studies of the motor system have historically used single pulse or short duration (<50 ms) stimulus trains composed of parameters proven to effectively elicit responses from the motor cortex [16]. These high frequency short duration trains of intracortical microstimulation (HFSD-ICMS) are typically used to map motor areas of the brain by observing the brief movements or recording the evoked muscle activity they produce in anesthetized animals [17–22]. The study of corticomotoneuronal cell activity can be used to predict electromyographic (EMG) activity [23]. Likewise, EMG activity recorded while stimulating the motor cortex provides insight into the relationship between the activity of cortical neurons and motor outputs [24]. Applying electrical stimulation to the forelimb region of the rat motor cortex activates corticospinal neurons and consequently activates the motor neurons innervating the forelimb muscle fibers producing contractions which can be recorded through EMG.

The precise mechanism in which electrical stimulation activates neural pathways to evoke responses is not completely known, particularly with respect to the type of cells activated and the volume of tissue recruited. Previous modeling and electrophysiology studies have suggested that stimulation with symmetric waveforms activates axons resulting in a greater volume of tissue activated, whereas asymmetric waveforms activate cell bodies producing more localized activation [25–29]. Stimulation with long duration trains may also result in greater volumes of tissue activation due to multi-synaptic projection.

Some general effects of stimulation parameters have been explored using short stimulus trains [30–32]. Stimulus frequency and train duration exert a combined influence on threshold levels by facilitating muscle contractions when excitation is hindered by low current intensity. Thresholds can be lowered by extending the train duration beyond 30 ms and increasing the pulse frequency above 300 Hz [16,33], and the lowest movement thresholds occur when stimulating with frequencies between 181–400 Hz for durations of 15–33 ms [34]. Independently, the parameter of stimulus frequency can be used to limit the spread of the ICMS signal within the cortex. Pulses delivered at frequencies less than 20 Hz prevent the summation of excitatory and inhibitory postsynaptic potentials which localizes the activation [35]. Stimulus train duration is the dominant parameter influencing the accuracy of forelimb movement trajectories. Stimuli which last for 500–1000 ms are known to generate forelimb movements to stable, predictable end points regardless of initial limb position [36].

To better understand the mechanism of microstimulation, it is essential to determine the exact effect exerted by each stimulus parameter on the resulting cortical activation and consequently, the outputs driven by this activity. Previously, we explored the influence of stimulation parameters on the amplitude and latency of evoked responses [37]. Other studies have examined the effects of certain parameters on threshold levels [38–43], however the duration of the EMG signal has been widely ignored. To our knowledge, the temporal components of the response have not been assessed. Here we systematically explore the effect of each parameter of a stimulation signal on the duration of the motor evoked potentials (MEP) elicited by microstimulation of the caudal forelimb area (CFA) of the rat primary motor cortex (M1). We assess the duration of the electrographic response evoked by the stimulus train and discuss the implications of response duration as an assessment parameter.

## Methods

### Surgical Procedures and Data Collection

Seven female Sprague-Dawley rats (Charles River, QC, CA) weighing 273–450 g were used in terminal acute experiments. All procedures followed the guidelines of the Canadian Council on Animal Care and were approved by the Comité de Déontologie de l'Expérimentation sur les Animaux of the Université de Montréal.

Anaesthesia was induced with intraperitoneal injection of ketamine (80 mg/kg) and xylazine (10 mg/kg) and maintained with isofluorane (~2% in 100% oxygen). Subcutaneous injection of mannitol (4 g/kg) and intramuscular injection of dexamethasone (1 mg/kg) were given prior to the craniotomy to prevent swelling and oedema. A self-regulating heating pad maintained body temperature which, along with pulse rate and oxygen saturation, was monitored continuously throughout the surgery. Insulated, multi-stranded wires (Cooner Wire, Chatsworth CA, USA) were implanted in the *extensor digitorum communis* (EDC) muscle of the forelimb contralateral to the stimulating electrode to detect MEP signals which were monitored and recorded at 5 kHz (RZ5 BioAmp Processor) and analyzed offline.

The animal was placed in a stereotaxic frame for both the surgical and stimulation procedures; positioned to allow free movement of the forelimb. A small craniotomy (8 mm x 5 mm) exposed the motor cortex (left hemisphere), the dura was removed and mineral oil applied to protect the cortex. After the surgery, gas anaesthesia was stopped and the animal was sedated with ketamine administered through intraperitoneal injections as needed (~10 mg/kg/10 minutes) in response to the state of the animal, which was closely monitored for the duration of the data collection procedure.

In order to control for the effects of stimulus current amplitude and ensure a consistent level of excitability across all test sites, we set site selection criteria. Sites were chosen within the caudal forelimb region of the motor cortex that produced MEP responses in the EDC. According to our previous cortical mapping experiments in Sprague-Dawley rats of comparable age and size [44–46], all sites were located at stereotaxic location approximately corresponding to the middle of the CFA. Exploratory mapping was used to determine MEP threshold levels at these sites by increasing the stimulus amplitude up to 50 μA until a MEP response was observed. We then lowered the stimulus amplitude until the response disappeared. The lowest stimulus amplitude at which the MEP response could be evoked was defined as the threshold amplitude.

Sites within the CFA were tested for the selection criteria using a standard ICMS train: 13 monophasic square pulses of 0.2 ms duration with 3.3 ms between the pulses delivered at 1 Hz [18,21,44,47]. The response was first tested at 1500 μm targeting output layer V, which is known to contain pyramidal neurons which project to lower motor neurons to produce movements [48]. If a threshold MEP response was produced in the EDC this depth was selected, if not, the electrode was advanced to find a suitably responsive site. Electrode depth varied from 1534–2104 μm (mean 1792 μm) between sites and only one depth was used within an electrode tract. All experimental blocks were tested at 2 sites per rat.

In order to provide a reference for each parameter used in the study we set ‘control’ levels within each parameter range based on the standard ICMS train [18,21,44,47]. As such, the control value for stimulus pulse duration was set to 0.2 ms, the control value for frequency was set to 303 Hz (corresponding to 3.3 ms between pulses), and the control value for train duration was set to 43 ms (corresponding to 13 pulses of 0.2 ms duration delivered with 3.3 ms between pulses). In order to set a control value for amplitude, we chose a value twice as large as the site’s threshold amplitude. We sought stimulation sites which had MEP threshold amplitudes of 25–35 μA to ensure that all test sites had similar levels of excitability and thus set the control value for amplitude to 50 μA in order to be twice as strong as the site’s threshold level. The control values and parameter ranges are detailed in Table 1.

**Table 1:** Parameter Test Values.

Parameter	Unit	Range	Test Levels	Control	Parameter Pairs
Amplitude (A)	μA	30–65	30, 39, 48, 56, 65	50	AF, AP, AI, AT
Frequency (F)	Hz	100–500	100, 200, 300, 400, 500	303	FA, FP, FI, FT
Pulse Duration (P)	ms	0.18–0.5	0.18, 0.26, 0.34, 0.42, 0.5	0.2	PA, PF, PI, PT
Interphase Interval (I)	ms	0.08–0.5	0.08, 0.19, 0.29, 0.40, 0.5	0	IA, IF, IP, IT
Train Duration (T)	ms	43–300	43, 107, 172, 236, 300	43	TA, TF, TI, TP

Stimulation was delivered with a digital stimulator (TDT IZ2 Stimulator and RZ5 BioAmp processor), through a glass insulated tungsten microelectrode (FHC Bowdoin, ME USA, UEWSDESGBN4G, 110–175 kΩ) manipulated by a microdrive (David Kopf Instruments Model 2662, Tujunga, CA). At the end of the data collection, the animal was euthanized with a lethal dose of sodium pentobarbital.

### Stimulation Protocol

**Fig 1:**
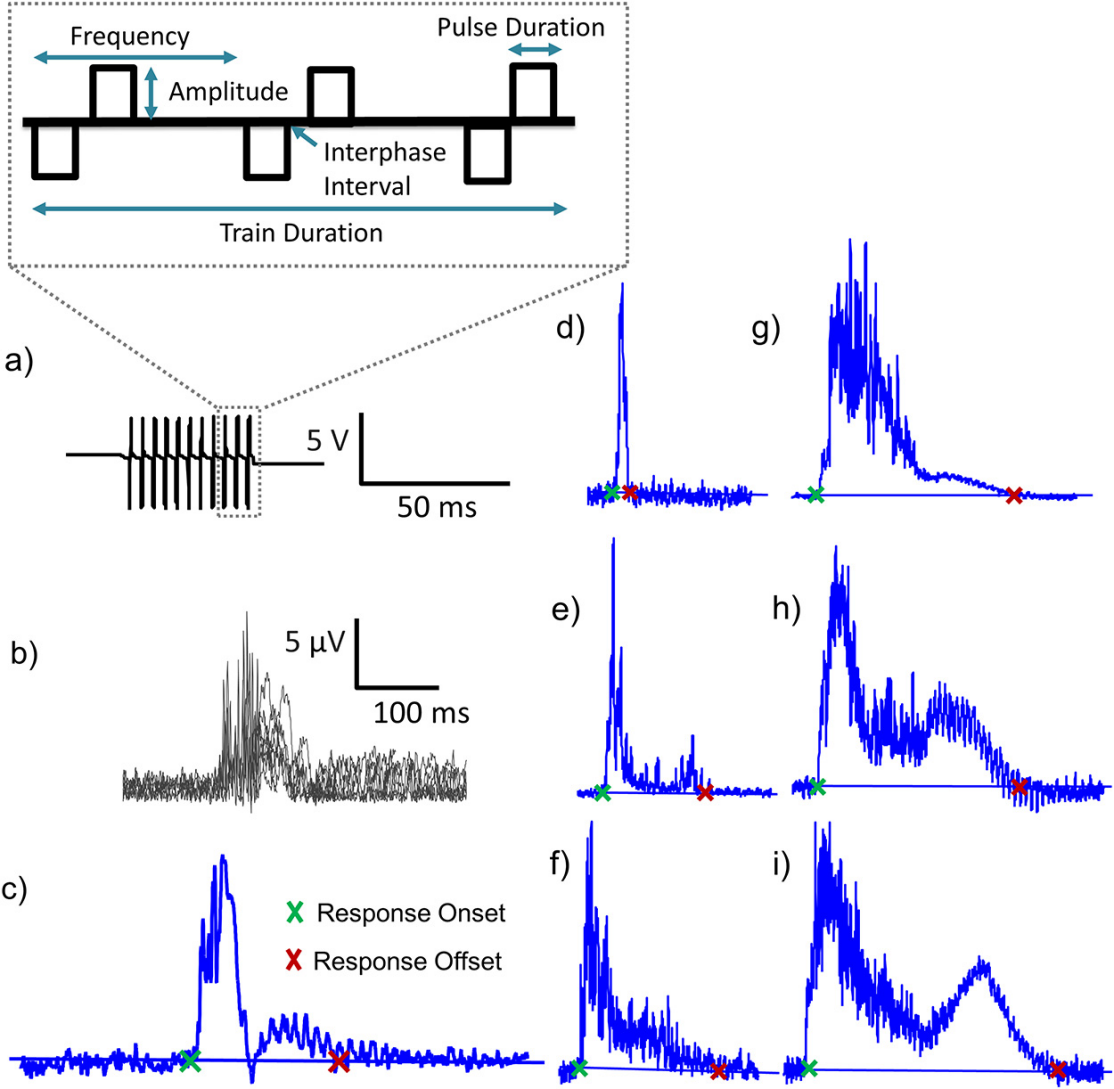
Stimulation signal and motor evoked potential responses. Part (a) depicts the parameters of the constant-current, biphasic square waveform stimulus. Part (b) depicts the MEP onset and offset definition and the scale in part b applies to parts b-i. Parts b-i demonstrate that a variety of signal envelopes were evoked by the stimulus parameter ranges tested. The duration of the response was determined by subtracting the signal onset time from the offset time. The response onset was defined as the first instance where ten sequential sample points of the MEP signal remained above the trial’s baseline level. Similarly, the response offset was the last instance in which the MEP signal returned to the baseline level and remained there until the end of the trial.

We designed a stimulation protocol to systematically test the influence that each parameter of an ICMS signal exerted on the MEP it produced when delivered to the CFA of the rat motor cortex. The constant-current, cathode leading, biphasic square waveform was chosen since it is the most prevalent in both research and therapeutic applications of ICMS. The parameters of this signal include the current amplitude, pulse frequency, pulse duration, interphase interval and pulse train duration (Fig 1a). We selected the test ranges of each stimulation parameter so that they included the typical values used in common prosthetic devices and therapeutic applications of brain stimulation. In particular, the ranges reflect the most restrictive stimulation paradigm among the applications: visual prosthetic devices [2].

The test range of each parameter was divided evenly into five levels (low, low-mid, mid, mid-high, and high) and a control value was set which was derived from the standard stimulation signal proven to be effective in the rat motor cortex as described above [18,21,44,47]. The control value for amplitude was set to 50 μA, which was twice the threshold level since all stimulation sites were deliberately chosen to have thresholds of approximately 25 μA. The ranges, levels and control values selected for each parameter can be found in Table 1.

The stimulation protocol was composed of five experimental blocks designed to test the specific influence that each of the five parameters of the stimulation signal exerted on the MEP signal. Each block focused on one parameter, systematically testing it against the other four parameters in a pair-wise fashion. The parameter of focus was called the primary parameter and each parameter tested against it was referred to as the paired parameter. The primary parameter was tested at all five levels in the range (low, low-mid, mid, mid-high, and high) against three levels of a paired parameter (low, mid, high) while all other parameters were held at their control values (see Table 1). This allowed us to observe how the MEP signal changed in response to changes in the primary parameter and identified interactions occurring between the primary and paired parameters.

To test the effects of current amplitude for example, all five values in the amplitude range (30, 39, 48, 56, 65 μA) were tested at 3 frequency levels (low-100 Hz, mid-300 Hz, and high-500 Hz) with pulse duration, interphase interval and train duration held at the control values (0.2 ms, 0 ms and 43 ms respectively). Similarly, all five values in the amplitude range (30, 39, 48, 56, 65 μA) were tested at 3 pulse duration levels (low-0.18 ms, mid-0.34 ms, and high-0.5 ms) with frequency, interphase interval and train duration held at the control values (303 ms, 0 ms and 43 ms respectively). This procedure was then repeated until each parameter had been tested against amplitude in this “pair-wise” arrangement representing all the conditions contained in the amplitude test block. All “parameter pairs” are listed in the far right column of Table 1.

Each pair-wise condition within a block was tested with ten trials, and all trials within a block were pseudo-randomized with 1 second between trials. The trial order within a block was preserved, and to ensure that there were no adverse effects of conducting the trials of a block in a fixed order we compared results from overlapping conditions between blocks. These comparisons were also used to determine if the primary parameter of the block had an overall effect on the MEP signals produced within it. The five experimental blocks resulted in a total of 300 independent test conditions (5 parameters x 5 test levels x 4 paired parameters x 3 test levels). To control for fatigue of the preparation, all five blocks were tested in a randomized order at two different sites within the CFA of each rat for a total of 14 sites.

### Classification of MEP Response Duration

The application of a biphasic square wave stimulus (Fig 1a) evoked a wide variety of MEP signal envelopes in response to the different parameter combinations (Fig 1b–1i). The responses could resemble a single Gaussian shaped curve (Fig 1d), or an initial Gaussian shaped curve followed by a secondary component of lower amplitude which can take the shape of a Gaussian curve (Fig 1e, 1h and 1i), exponential decay (Fig 1f), or inverse exponential decay (Fig 1g). This secondary component can be entirely absent (Fig 1d) or endure for hundreds of milliseconds.

The duration of the response was determined by subtracting the signal onset time from the offset time. From a computational standpoint, a number of criteria were developed to automate the designation of the response onset and offset. The MEP response’s baseline was defined as the average of the recorded signal taken 10 ms prior to stimulation. Averaging the first 50 sample points of the recording provided the baseline for each individual trial. The response began shortly after the onset of stimulation, and was detected as the first instance where ten sequential sample points of the MEP signal remained above the trial’s baseline level. Similarly, the response offset was the last instance in which the MEP signal returned to the baseline level and remained there until the end of the trial. The response onset and offset times were determined computationally and their accuracy was confirmed with visual inspection.

### Statistical Analyses

A maximum of two trials per condition were excluded if they were contaminated by noise. The stimulus artifact was relatively small due to the low amplitudes of stimulation and was removed with a 5-point moving average filter. For each of the 14 stimulation sites, trials were averaged to produce a mean response for each condition. The mean of the response duration was derived from these averages (Fig 1) and values are reported as mean ± standard deviation.

Certain combinations of parameter pairs using current amplitudes near threshold levels did not cause sufficient excitation to evoke any MEP responses. Specifically, 30–39 μA stimuli delivered at 100 Hz, or 30 μA stimuli delivered by 43 ms duration trains were ineffective. A condition was included in the statistical analyses if responses were obtained from a minimum of 5 stimulation sites. Conditions evoking a response from less than 5 sites will be referred to as infrequent responses and typically occurred for stimuli composed of low amplitude short pulses delivered at low frequencies. To differentiate between absent and infrequent responses, data obtained from infrequent responding conditions (<5 sites) are included in graphical representations differentiated by marker type.

To evaluate the effects of each parameter on the response metrics we used a repeated measures linear mixed model, with random slope and intercept, followed by Bonferroni posthoc analyses to test the effects of main and paired parameters on outcomes. Main and paired parameters were entered as fixed factors, followed by the interaction between the two. The interaction term was not significant and was trimmed from the model. This model compared the trends of each factor to quantify differences in response duration evoked by the tested parameter levels (low, mid, high). Analyses were conducted with SPSS 22.0 (SPSS, Inc.) software using the mixed procedure with a factorial design and a significance level of α<0.05. We report only statistically significant findings. If a value is absent it indicates that the particular condition either did not produce significant trends or was excluded from the analyses due to infrequent responses (<5 sites). In the text we indicate which results are excluded due to infrequent responses. Measures of percent increase in response duration between stimulus levels were computed from estimated marginal means generated in the linear mixed model analysis. Correlation analyses between the duration of the MEP and other characteristics of the response were conducted with Pearson’s correlations. A *t* statistic was used to establish if the correlation was statistically significant.

## Results

**Fig 2:**
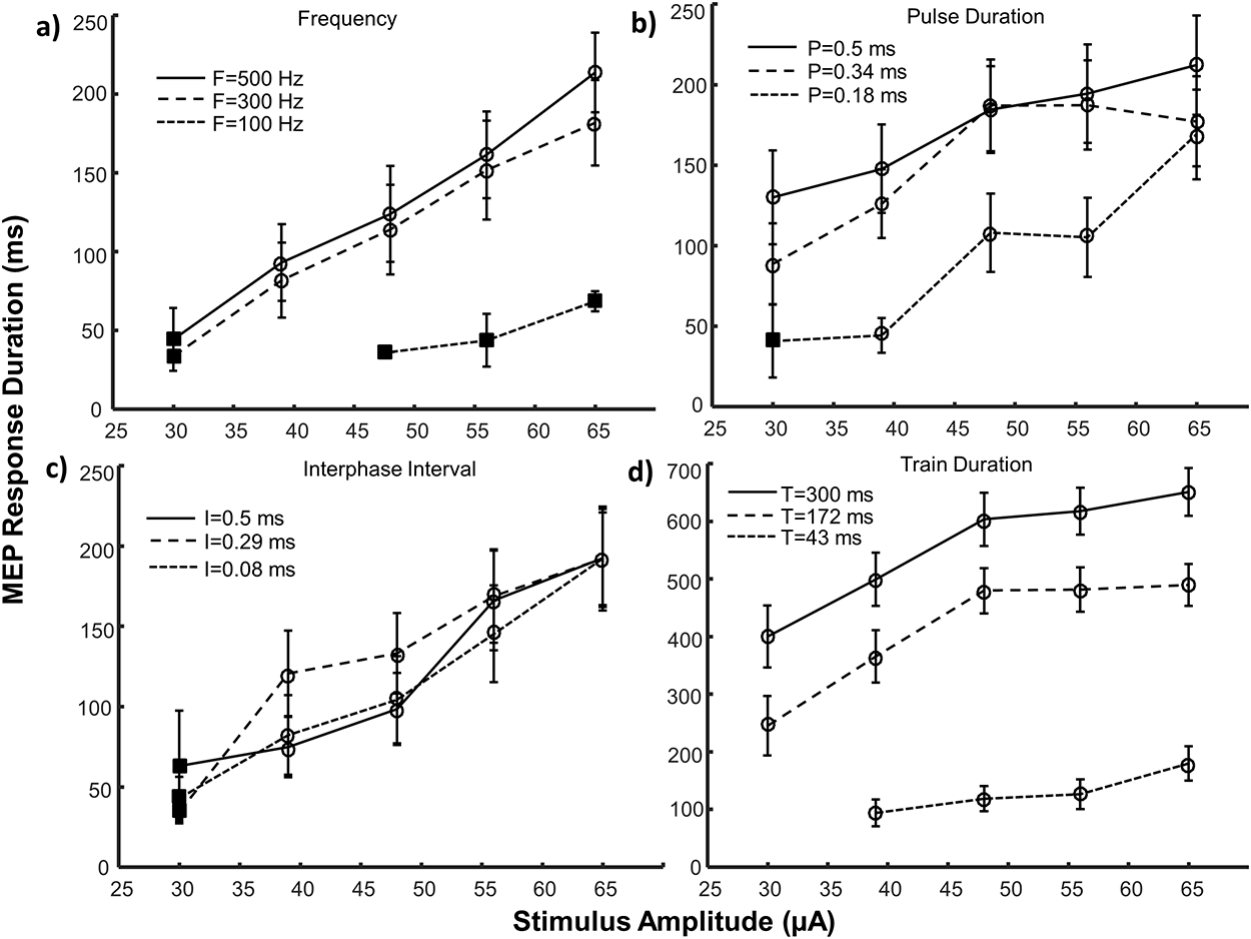
MEP main response duration (mean ± SE) as a function of stimulus amplitude. The effects of amplitude paired with three frequency levels (a), pulse durations (b), interphase intervals (c) and train durations (d) are depicted. Note the difference in scale for trials involving train duration (part d). Square symbols represent conditions with an insufficient number of responding sites (n<5) and were not included in statistical analyses. Circular symbols represent conditions with reliable responses (n = 5–14). Control values for each parameter were: A = 50 μA, F = 303 Hz, P = 0.2 ms, I = 0 ms, T = 43 ms. A = amplitude, F = frequency, P = pulse duration, I = interphase interval, T = train duration, SE = standard error.

**Fig 3:**
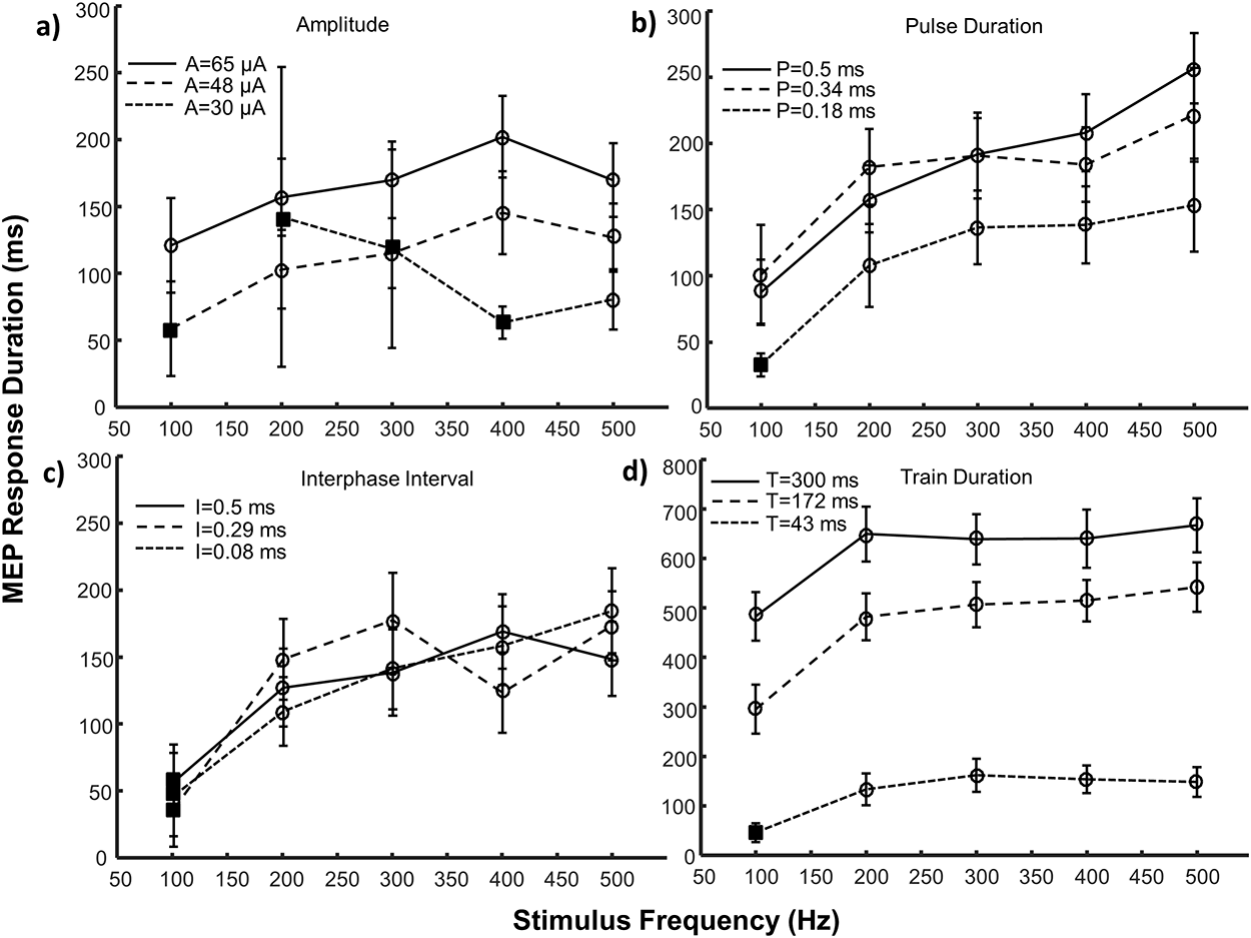
MEP main response duration (mean ± SE) as a function of stimulus frequency. The effects of frequency paired with three amplitude levels (a), pulse durations (b), interphase intervals (c) and train durations (d) are depicted. Note the difference in scale for trials involving train duration (part d). Square symbols represent conditions with an insufficient number of responding sites (n<5) and were not included in statistical analyses. Circular symbols represent conditions with reliable responses (n = 5–14). Control values for each parameter were: A = 50 μA, F = 303 Hz, P = 0.2 ms, I = 0 ms, T = 43 ms. A = amplitude, F = frequency, P = pulse duration, I = interphase interval, T = train duration, SE = standard error.

**Fig 4:**
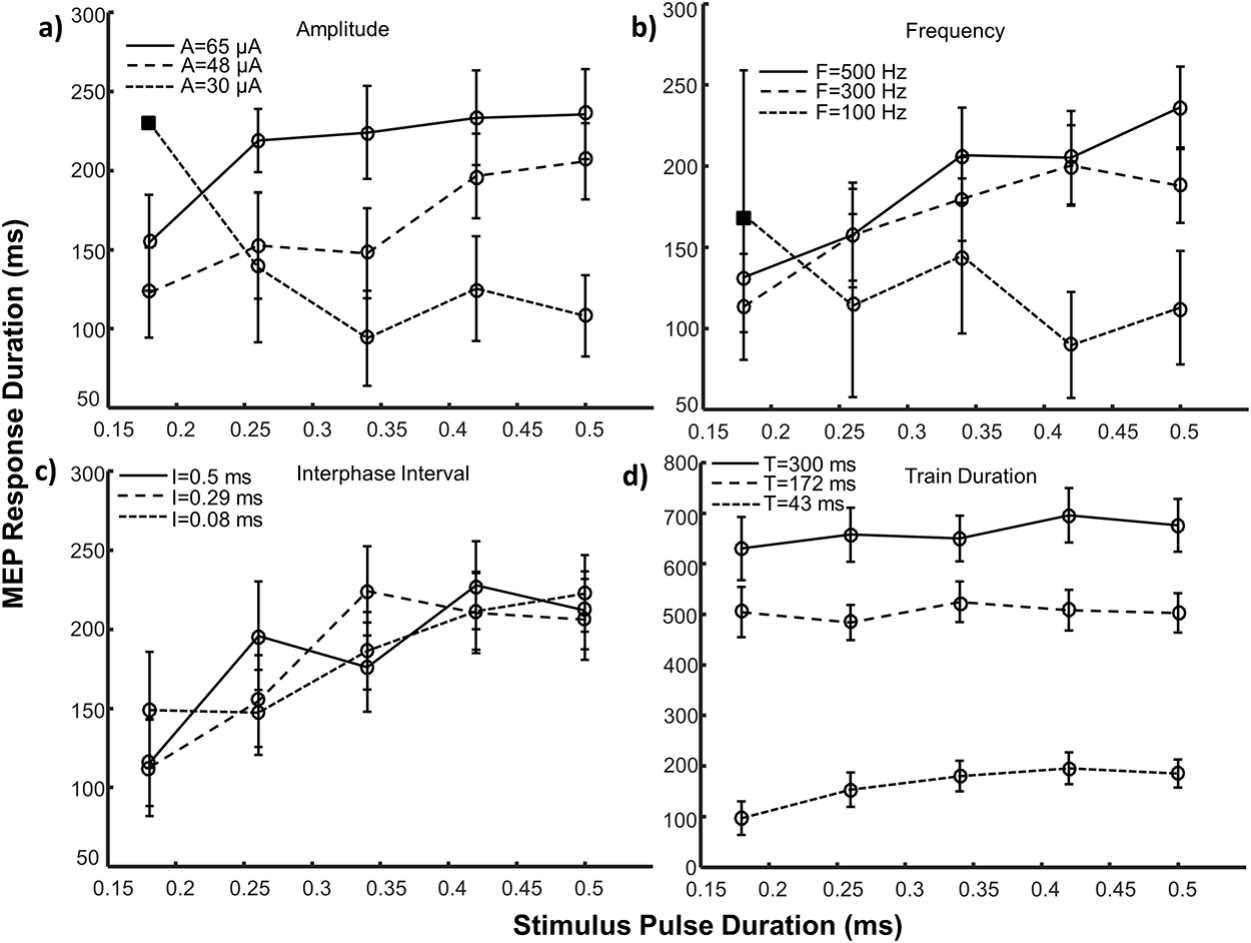
MEP main response duration (mean ± SE) as a function of stimulus pulse duration. The effects of pulse duration paired with three amplitude levels (a), frequencies (b), interphase intervals (c) and train durations (d) are depicted. Note the difference in scale for trials involving train duration (part d). Square symbols represent conditions with an insufficient number of responding sites (n<5) and were not included in statistical analyses. Circular symbols represent conditions with reliable responses (n = 5–14). Control values for each parameter were: A = 50 μA, F = 303 Hz, P = 0.2 ms, I = 0 ms, T = 43 ms. A = amplitude, F = frequency, P = pulse duration, I = interphase interval, T = train duration, SE = standard error.

**Fig 5:**
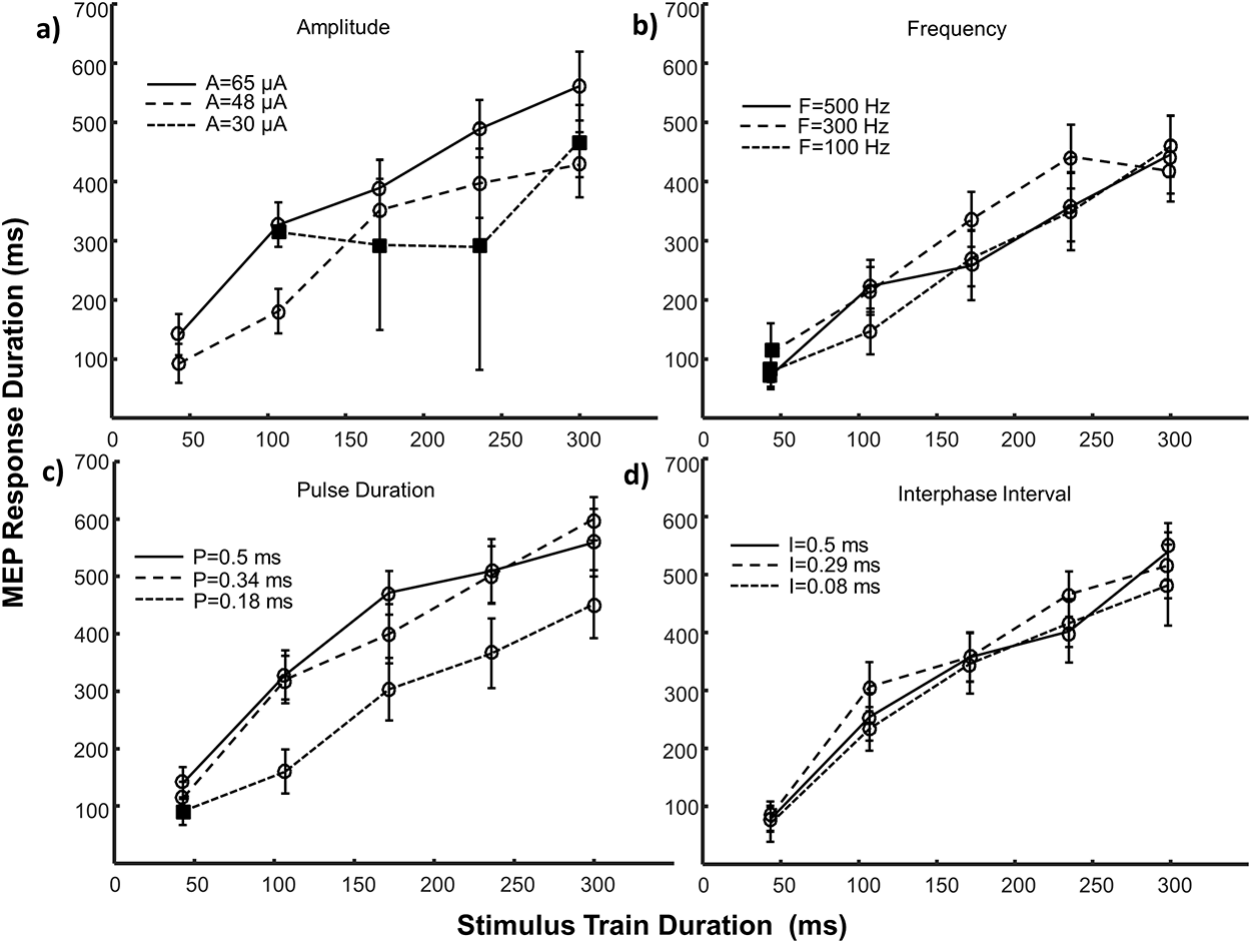
MEP main response duration (mean ± SE) as a function of stimulus train duration. The effects of train duration paired with three current amplitudes (a), frequencies (b), pulse durations (c) and interphase intervals (d) are depicted. Square symbols represent conditions with an insufficient number of responding sites (n<5) and were not included in statistical analyses. Circular symbols represent conditions with reliable responses (n = 5–14). Control values for each parameter were: A = 50 μA, F = 303 Hz, P = 0.2 ms, I = 0 ms, T = 43 ms. A = amplitude, F = frequency, P = pulse duration, I = interphase interval, T = train duration, SE = standard error.

**Fig 6:**
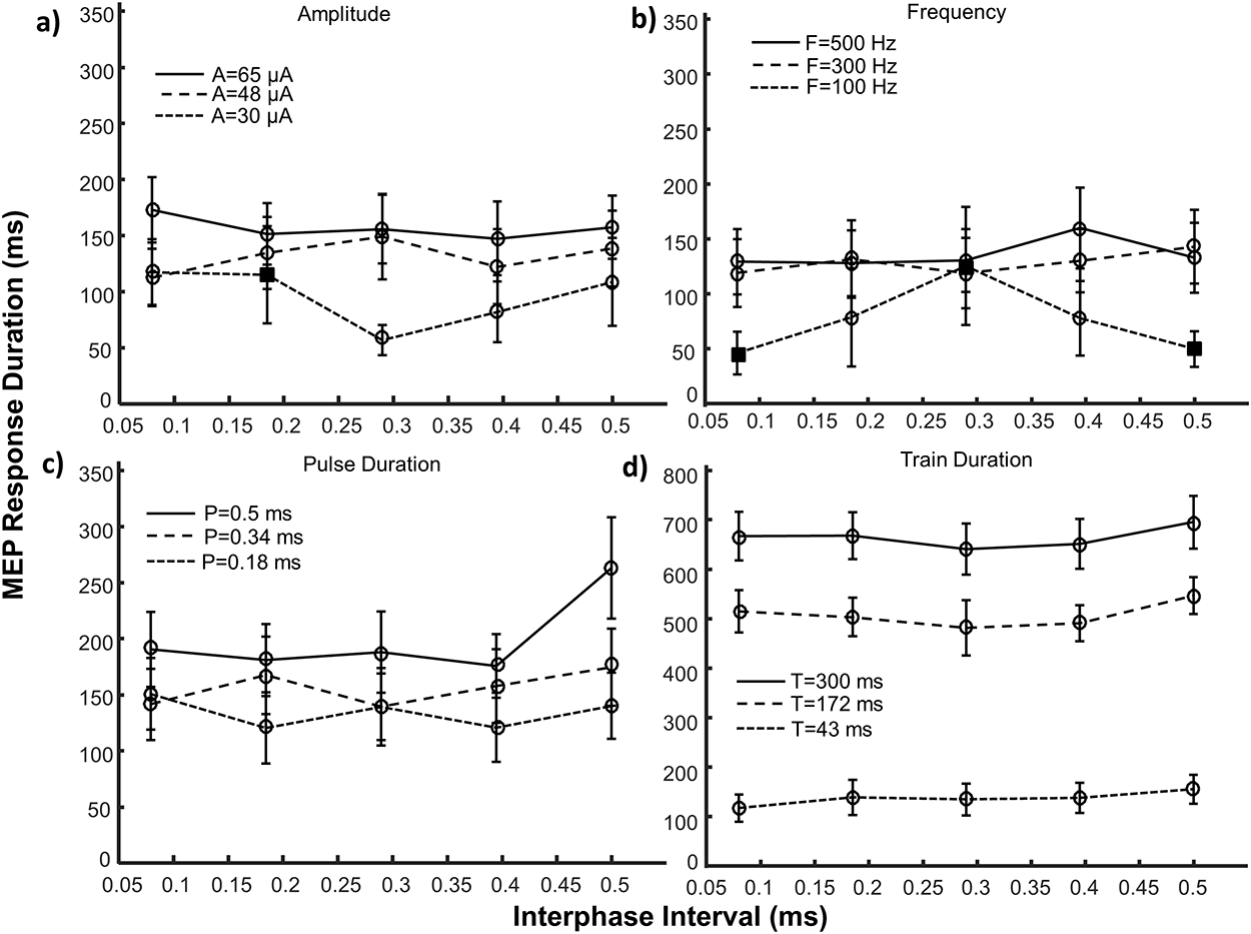
MEP main response duration (mean ± SE) as a function of stimulus interphase interval. The effects of interphase interval paired with three amplitude levels (a), frequencies (b), pulse durations (c) and train durations (d) are depicted. Note the difference in scale for trials involving train duration (part d). Square symbols represent conditions with an insufficient number of responding sites (n<5) and were not included in statistical analyses. Circular symbols represent conditions with reliable responses (n = 5–14). Control values for each parameter were: A = 50 μA, F = 303 Hz, P = 0.2 ms, I = 0 ms, T = 43 ms. A = amplitude, F = frequency, P = pulse duration, I = interphase interval, T = train duration, SE = standard error.

Four of the five experimental blocks showed significant trends demonstrating the effects that each parameter exerted on the MEP response duration. The interphase interval parameter did not exert an influence on the MEP response duration within the range tested (0.08–0.5 ms) and therefore will not be described beyond graphical depiction as a paired parameter in Figs 2c, 3c, 4c, 5d and 5a main parameter in Fig 6a–6d. These findings are included to present a complete picture of the parameter interactions and negative results can often be informative. When describing the results, all parameter pairs are denoted by abbreviations of Table 1 in which the primary parameter appears first and paired parameter second (ex. AF represents amplitude paired with frequency).

### MEP Response Duration

The MEP response duration increased with stimulus amplitude for all parameter pairings (AF: p<0.001; AP: p<0.001; AT: p<0.001) as shown in Fig 2. As a paired parameter (Figs 3a, 4a and 5a), increases in stimulus amplitude could extend the response duration up to 48% when its value was raised from its mid (48 μA) to high (65 μA) levels (FA: 48% increase, p = 0.037; PA: 30% increase, p = 0.009; TA: 30% increase, p = 0.001) and 68% when raised from low (30 μA) to mid (48 μA) levels (PA: 68% increase, p = 0.003). When high amplitude (48–65 μA) stimuli were delivered by long duration trains the response duration plateaued (Fig 2d) suggesting an upper limit for this parameter pairing after which the duration of the response cannot be extended. Conversely, when low amplitude stimuli are used, the duration of the response is most effectively extended by increasing the stimulus pulse duration or train duration (Fig 2b and 2d).

The MEP’s response duration increased with stimulus frequency for all parameter pairings (FP: p<0.001; FT: p = 0.005), except amplitude (FA: p = 0.140), as shown in Fig 3. When stimulating with frequencies above 200 Hz, the response plateaued at a maximum whose magnitude was dictated by the paired parameter. Increasing the frequency from low (100 Hz) to mid (300 Hz) levels could extend the response duration (PF: 79%, p = 0.006), however mid (300 Hz) and high (500 Hz) frequency stimuli evoked similar durations (AF: p = 0.39; PF: p = 0.68; TF: p = 1.0). When stimulation frequency was low, extending the duration of the stimulus train was the most effective method of increasing the response duration (Fig 3d).

The MEP response duration increased with stimulus pulse duration except when paired with train duration (PA: p = 0.007; PF: p = 0.002; PT: p = 0.06), as shown in Fig 4. Increasing stimulus pulse duration from short (0.18 ms) to mid (0.34 ms) levels extended the duration between 39–82% (AP: 82%, p<0.001; FP: 43%, p = 0.025; TP: 39%, p = 0.002), however no significant differences were noticed between mid (0.34 ms) and long (0.5 ms) duration pulses (AP: p = 0.626; FP: p = 1.0; TP: p = 0.965). When short pulse durations were used, the response duration was most effectively extended by increasing the train duration.

The MEP response duration increased with stimulus train duration for all parameter pairings (TA: p<0.001; TF: p<0.001; TP: p<0.001), as shown in Fig 5. These effects proved to be less pronounced statistically once the train duration reached 172 ms and visually this change could possibly occur even earlier at 107 ms (Fig 5). As a paired parameter, increasing stimulus train length from short (43 ms) to mid (172 ms) durations could extend the response duration up to 3.5 times (AT: increase 3.50x, p<0.001; FT: increase 3.50x, p<0.001; PT: increase 3.06x, p<0.001).

Similarly, increasing the train length from mid (172 ms) to long (300 ms) durations increased the response duration yet again (AT: increase 1.32x, p<0.001; FT: increase 1.28x, p<0.001; PT: increase 1.31x, p<0.001). For all durations of the stimulus train, increasing the amplitude or pulse duration served to further extend the duration of the MEP’s response; however pulse duration had less effect on short duration trains (43 ms). A summary of these findings is presented in Table 2.

**Table 2:** Summary of Parameter Influence on Response Duration.

Parameter	Effect on Response Duration (RD)
Amplitude	RD increased with stimulus amplitude for all parameter pairings except interphase interval 68% increase in RD between low and mid amplitude stimuli 48% increase in RD between mid and high amplitude stimuli RD reached a plateau when high amplitude stimuli delivered by long trains RD for low amplitude stimuli most effectively increased by extending stimulus pulse or train duration
Frequency	RD increased with stimulus frequency for all parameter pairings except stimulus amplitude and interphase interval 79% increase in RD between low and mid frequency stimuli No significant difference in RD between mid and high frequency stimuli Stimulating at frequencies higher than 200 Hz caused the RD to plateau RD for low frequency stimuli most effectively increased by extending the duration of the stimulus train
Interphase Interval	No effect
Pulse Duration	RD increased with stimulus pulse duration for all parameter pairings except stimulus train duration and interphase interval Up to 82% increase in RD between short and mid duration pulses No significant difference in RD between mid and long pulse duration stimuli RD for short pulse stimuli most effectively increased by extending the train duration
Train Duration	RD increased with stimulus train duration for all parameter pairings except interphase interval 3.5x increase in RD between short to mid duration trains 1.3x increase in RD between mid and long duration trains Effects of train duration on RD were less pronounced for trains >172 ms Increasing amplitude or pulse duration could extend RD for all train lengths

### Correlation between Duration and Other MEP Parameters

Certain metrics of the MEP response were valid predictors of the response duration. A total of four metrics in addition to the duration served to quantify the MEP response. Onset latency was defined as the delay between the onset of stimulation and the initiation of the MEP. The mean was computed as the average of the response, whereas peak amplitude was the signal’s maximum during the response. Peak time specifies the time instance of the peak amplitude occurrence. Pearson’s correlation coefficient was computed for each block of trials to provide a quantitative measure of the strength and direction of the relationships between the response metrics [49]. The response duration was not correlated with onset latency (r = 0.05, p>0.05) but was strongly correlated with peak amplitude (r = 0.49, p<0.001), mean amplitude (r = 0.56, p<0.001) and peak time (r = 0.63, p<0.001).

## Discussion

In our previous study, we examined the factors influencing the amplitude and latency of the MEP response and defined methods for modulating these parameters [37]. We found that MEP amplitude increased continually with stimulus amplitude and train duration; however both frequency and train duration exhibited upper limits after which no further effects on the response were observed. The MEP onset latency was found to decrease continually with increases in pulse duration; however both amplitude and frequency observed upper limits after which they became ineffective and train duration demonstrated no influence on the response latency. The present study examined the factors influencing the duration of the MEP response in the intact CNS, defined methods for modulating the response duration, and identified correlations between the spatial and temporal metrics of the MEP. We observed that responses evoked by low amplitude stimuli are best extended by increasing the stimulus pulse duration or train duration. Similarly, responses to low frequency or short pulse duration stimuli can be prolonged by extending the duration of the stimulus train. However, increases in amplitude or pulse duration serve to extend the response duration for all lengths of stimulus train. These findings may be used as modulation techniques when designing stimulation signals within restrictive paradigms. Together with our previous results [37,50,51], we demonstrate that all stimulus parameters, with the exception of interphase interval, exert a significant influence on the motor responses they evoke.

We observed a lack of influence of the interphase interval parameter on the duration of MEP responses suggesting that the delay between pulses of alternate polarity does not significantly influence the evoked responses. Alternating the polarity of the phases in the constant-current, biphasic square waveform is a method used to balance the charge injected into the tissue [52]. The effects of the charge exerted in the initial phase are mitigated by stimulating with the opposite polarity in the second phase, and the charge is said to be “recovered” through this alternation of polarity. Extending the interval between the two phases is thought to delay the charge recovery and makes the biphasic stimulus behave more like its monophasic counterpart, which can evoke responses at lower thresholds by allowing charge to accumulate in the tissue. Extending the interphase interval of a biphasic stimulus has been shown to lower thresholds for phosphene induction in the visual cortex of human subjects [2], as well as for excitation of the auditory nerve in cochlear implants [53]. However, studies that manipulated pulse phase symmetry, which is an alternate method of delaying charge recovery, were unable to alter threshold levels for ICMS in rodents [38].

Our findings demonstrated that the shape of the MEP response signal envelope varies greatly and is heavily dependent on the stimulation parameters; however certain temporal and spatial metrics of the MEP response could be used to predict the duration of the response. Of the temporal metrics, only peak time was a strong predictor of the response duration; the later the peak time, the longer the overall duration of the MEP. Of the spatial metrics, both the peak and mean amplitudes were strongly correlated with the response duration. These findings suggest that the spatial and temporal metrics of the MEP response are directly linked and their interactions must be considered when designing a stimulus signal.

We demonstrated that the duration of the MEP response can be modulated by altering parameters of the stimulation train. The MEP response duration increased with all stimulus parameters (except interphase interval) and this increase was continual for the parameters of stimulus amplitude and train duration although the effects were less pronounced for trains longer than 172 ms. Our experimental design involved the comparison of low, mid and high levels of each parameter, in the case of train duration these values are 43, 172 and 300 ms respectively. As such, it is possible (and suggested from Fig 5) that the reduction in influence occurs earlier than 172 ms, possibly occurring at 107 ms. Notably, the MEP durations did not scale directly with the duration of the stimulus train and responses as short as 25 ms could be evoked with a 43 ms stimulus. For the parameters of frequency and pulse duration, no further increases in response duration were observed beyond the range of 100–200 Hz and 0.18–0.34 ms respectively.

Previous studies reported the lowest movement thresholds to occur when stimulating with frequencies between 181–400 Hz, with no significant difference in thresholds for frequencies between 142–400 Hz [34]. Our previous work demonstrated that increasing the stimulus frequency higher than 100–200 Hz did not exert further influence on MEP reliability [37,50]. Combined with our present results we suggest that the rat forelimb motor cortex is sensitive to frequencies below 142 Hz and no further excitation is produced by the higher frequency stimulation commonly used in this system. If generalized, this finding could serve to further optimize the standard ICMS signal used in the study of the motor cortex. This signal has historically involved stimulation frequencies of 303 or 333 Hz [18,21,44,47], which our data suggest is above the range for which frequency exerts an influence on the production of MEPs. Reducing the frequency of stimulation to 142 Hz could potentially allow for more precise definition of the somatotopic boundaries between cortical representations of different movements identified by motor mapping techniques. For example, the use of lower frequency stimulation could perhaps reduce the occurrence of dual responses (i.e. two simultaneously evoked movements at threshold stimulation intensity), such as those occurring near the boundary of the forelimb and whisker representations of the rat motor cortex.

Similarly, studies exploring the cortex with long duration stimulation (HFLD-ICMS) typically use 500 ms trains [36,43,54,55], which are nearly 3 times the maximum level we observed to exert an influence on response duration and nearly 5 times the level we observed to influence MEP reliability, amplitude and onset latency [37,50]. The plateaus observed in our results suggest that the influence of the stimulus saturates before the offset of long duration trains. In light of these results it would be interesting to test whether or not shorter trains of durations intermediate to HFSD and HFLD could be capable of producing the same movement repertoires obtained through HFLD-ICMS stimulation.

These findings should be explored in other species and target locations (visual cortex/auditory nerve versus motor cortex) to confirm whether or not these relationships are preserved. For instance, it would be interesting to verify if they are similar in species with direct corticomotoneuronal connections such as macaques [56]. In addition, the present data was exclusively recorded in EDC. It is possible that other muscles, such as intrinsic hand or more proximal muscles could show different profiles of modulation. As such, the relationships demonstrated here should be confirmed in other muscles and species before generalization.

To our knowledge, the duration of the MEP response is not generally considered in the design of stimulation signals; however it may have significant implications. In applications where a brief, localized stimulus is desired, a prolonged duration of activation may not be desirable. An example application could be the generation of a punctate visual percept through application of electrical stimuli to the visual cortex [2]. In these instances, stimuli involving combinations of low amplitude, low frequency, short pulse durations and short train durations might be used to limit response durations. Conversely, when restrictions are placed on certain stimulus parameters and longer response durations are desired, the response duration can be increased most effectively by extending the stimulus train duration and to a lesser extent by increasing the pulse duration and amplitude. An example application might be reducing the required frequency of stimulation provided during deep brain stimulation for the treatment of Parkinson’s disease [11] or the prevention of epileptic seizures [12]. It is possible that our response modulation techniques may have an important impact on the efficacy of the stimulation for various applications. The extension of these trends to other application will require further study to verify that the influence of stimulus parameters is preserved between systems.

## Conclusion

Our findings demonstrate that the temporal properties of the evoked response in the intact CNS can be both *predicted* by certain response metrics and *modulated* via alterations to the stimulation signal parameters. Frequencies above 200 Hz do not improve the reliability or extend the duration of MEPs and stimulating with trains longer than 172 ms does not substantially extend the response duration. Short duration responses may suggest more localized stimulation and can be achieved by limiting the parameters of a stimulus, and long duration responses could suggest greater synaptic spread. As such, it is essential to consider the desired physiological response and appropriate parameter combinations necessary to achieve it when designing a stimulus, and both the properties and underlying cause of the variability in signal envelopes generated by stimulus trains deserve further study.
